# Non-Invasive Delivery of Therapeutics into the Brain: The Potential of Aptamers for Targeted Delivery

**DOI:** 10.3390/biomedicines8050120

**Published:** 2020-05-14

**Authors:** Bakhtiar Bukari, Rasika M. Samarasinghe, Jinjutha Noibanchong, Sarah L. Shigdar

**Affiliations:** 1 School of Medicine, Deakin University, 3216 Geelong, Victoria, Australia; bbukari@deakin.edu.au (B.B.); r.samarasinghe@deakin.edu.au (R.M.S.); jnoibanc@deakin.edu.au (J.N.); 2 Centre for Molecular and Medical Research, Deakin University, 3216 Geelong, Victoria, Australia

**Keywords:** antibody, aptamer, blood-brain barrier, brain pathology, drug delivery, non-invasive, SELEX

## Abstract

The blood-brain barrier (BBB) is a highly specialised network of blood vessels that effectively separates the brain environment from the circulatory system. While there are benefits, in terms of keeping pathogens from entering the brain, the BBB also complicates treatments of brain pathologies by preventing efficient delivery of macromolecular drugs to diseased brain tissue. Although current non-invasive strategies of therapeutics delivery into the brain, such as focused ultrasound and nanoparticle-mediated delivery have shown various levels of successes, they still come with risks and limitations. This review discusses the current approaches of therapeutic delivery into the brain, with a specific focus on non-invasive methods. It also discusses the potential for aptamers as alternative delivery systems and several reported aptamers with promising preliminary results.

## 1. Blood-Brain Barrier

The blood-brain-barrier (BBB) is an interface that separates the contents of the blood circulatory system from the central nervous system (CNS) environment. It is made up of specialised vascular endothelial cells (EC) that regulate and restrict the movement of various compounds to and from the bloodstream to protect the cellular integrity and well-being of the brain and the CNS [1,2]. On the abluminal region, the BBB is lined with cells called pericytes, extracellular matrix protein known as basal lamina, and astrocytes. Intercellular features called tight junctions that are made up of proteins called occludin, claudin and junctional adhesion molecules, also contribute to the high selectivity of the BBB [1]. Additionally, structures known as fenestrae, or transcytoplasmic windows, are lacking on the BBB, which prevents rapid exchanges of intra- and extravascular solutes via paracellular transport between the blood and brain environments [3,4,5]. Together, all the components that make up the BBB interact with each other to modulate BBB function and characteristics [6,7,8,9].

**Figure 1:**
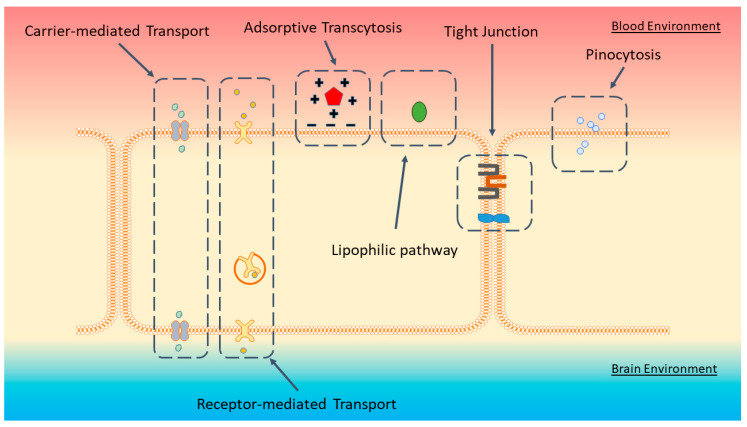
Summary of BBB anatomy and transport pathways. Passage of large molecules from the blood to the brain environments are limited to the carrier-mediated and receptor-mediated transport systems, adsorptive transcytosis, the lipophilic pathway, and pinocytosis. Tight junctions in between cells and the lack of fenestrations restrict free movement of molecules. Figure adapted from reference [6].

Several transcellular transport mechanisms have been known to take place on the BBB. These are passive diffusion by lipophilic substances, pinocytosis, carrier mediated transport (CMT) pathways, receptor mediated pathways (RMT) and adsorptive transcytosis (Figure 1) [10]. Of note, the CMT and RMT processes are mediated by highly specific transmembrane proteins, enabling the movement of only specific substances. Collectively, the unique features of the BBB EC, the surrounding cells, and extracellular matrix adjacent to them create a highly selective barrier that effectively separates the circulatory system from the brain environment. This is highlighted by the fact that only about 98% of small molecules (400–500 Da and below) can transcytose across the BBB, and 100% of large molecules are prevented from getting into the brain [11].

## 2. Delivering Therapeutics across the BBB

Due to its highly restrictive nature, the BBB presents challenges for the treatment of brain pathologies such as Parkinson’s disease, Alzheimer’s disease, as well as primary and secondary brain tumours. To get macromolecular drugs to reach the brain parenchyma, multiple approaches have been considered and studied extensively. Current strategies to deliver drugs into the brain can be broadly classified into two categories: invasive and non-invasive, each with their own advantages and limitations.

The most direct method of introducing therapeutics to the brain environment is via invasive means, which involves cutting or puncturing of the skin and/or internal organs, and insertion of instrumentations [12]. These approaches include intrathecal (IT) and intracerebroventricular injection, convection enhanced delivery (CED) and intracranial implantation. However, strategies such as IT and intracerebroventricular injections are not applicable to all brain pathologies especially with lesion sites that are embedded deep within the brain. Additionally, since the cerebrospinal fluid has little bulk flow, there are questions about therapeutic delivery efficiency into the brain via these injections [13]. CED involves the placement of catheters and infusion pumps that will improve therapeutic delivery into the brain using specially formulated infusate. The effectiveness of delivery and tissue distribution is however tied to therapeutic agents used, as reported by Hardy et al. in 2013 [14]. Moreover, a phase III trial on CED found that 68% of the catheters used are misplaced, which would have significantly impacted the method’s efficiency [15]. As the name suggests, intracranial implantation allows for the placing of therapeutic agents in direct contact with lesioned sites. However, the implantation procedure is very invasive and could lead to tissue damage and infection risks, which diminishes its safety profile [12,16]. Intranasal administration of therapeutics avoids the BBB altogether and instead relies on the olfactory and trigeminal nerve networks to transport therapeutics into the brain [17]. There are however several limitations to this method as therapeutic dosage is small (at about 100 µL), which necessitates multiple treatments, and targeted tissue delivery could not be achieved due to the branching pathways of the olfactory and trigeminal nerve networks [1,18].

Non-invasive therapeutics delivery via the circulatory system could be crucial in the treatment of brain pathologies in a less invasive approach as the microvessel network in the brain are very extensive such that no brain cells are more than ~10–25 µm away from a blood vessel [19,20]. Considering this, strategies have been studied and developed to circumvent the selective permeability of the BBB. Examples of BBB-crossing delivery strategies can be broadly divided into two categories: structural modification to increase BBB permeability (focus ultrasound, transient BBB disruption), and exploiting endogenous transport processes on the BBB to allow passage (cell-mediated delivery, cell penetrating peptide, nanoparticle, monoclonal antibody, and aptamer-mediated delivery).

### 2.1. Focus Ultrasound

In focus ultrasound (FUS), BBB disruption and therefore increased permeability is achieved by focusing a pulsed ultrasound beam at a targeted location with the aid of magnetic resonance imaging [21,22,23]. Permeability of the BBB is thought to be affected by acoustic beams leading to the temporary and reversible formation of fenestrations and channels in a process called sonoporation [24,25]. Preliminary studies have shown that horseradish peroxidase could successfully travel into the brain tissue via the transendothelial and paraendothelial pathways when injected intravenously following FUS treatment [26].

Although studies have been conducted suggesting its clinical safety and several phase I trials are currently underway, the caveat is that magnetic resonance guidance is a must to prevent inadvertent tissue damage from nonspecific ultrasound focusing [24,27]. Consequentially, this technique has the potential to be very costly to patients [24]. Furthermore, it should also be highlighted that this approach only assists in increasing the permeability of the BBB and as such could result in nonspecific entry of molecules or potentially pathogens as well.

### 2.2. Osmotic and Chemical Disruption of the BBB

With a more permeable barrier, macromolecular drugs or therapeutic carriers can travel across the BBB into the brain more effectively and at a higher rate. To achieve this, two main modes of BBB permeability modulation have been developed – osmotic and chemical disruption. Hyperosmotic opening of the BBB involves dilation of the blood vessels as well as shrinkage of cerebrovascular EC, leading to the widening of the tight junction to about 200 Å in diameter [1]. As a result, the permeability of the BBB increases tenfold for certain molecules, allowing them to pass through the BBB via paracellular transcytosis [28].

Joshi et al. however reported inconsistent BBB disruption in their study on intraarterial administration of mannitol in rabbits. Using Evan’s Blue dye, they showed that the duration and degree of BBB disruption varies across the brain tissue, and additionally, Chen et al. demonstrated that the treatment of mannitol did not increase BBB permeability in adult mouse forebrain [29,30]. For certain disease states such as ischemia, multiple sclerosis and inflammation, the BBB has already been dysregulated or affected by the pathologies [29,31]. As such, severe disruption induced by hyperosmotic agents could lead to more acute neuropathology such as seizures and brain oedema [29,31,32].

Chemical disruption of the BBB on the other hand utilises the ability of vasoactive agents to induce temporary inflammatory reactions in EC thereby increasing their permeability for up to 15 min [33]. Vasoactive agents such as bradykinin or its synthetic analogue, RMP-7, upon intra-arterial administration will bind to the B2 bradykinin receptor on the EC, increases cytosolic Ca^2+^ concentration which in turn activates nitric oxide production. Nitric oxide then promotes reversible vasodilation and enhanced EC permeability therefore allowing more molecules to pass through the BBB for a short period of time [24].

Even though a phase II clinical trial involving the treatment of recurring malignant glioma with carboplatin via RMP-7 induced BBB disruption showed promising results, unfortunately the same beneficial effect could not be replicated in a later trial stage, highlighting the lack of reproducibility when translated from early to late trials [24,34].

### 2.3. Cell-Mediated Delivery

Certain cells, such as immunocytes and stem cells, are naturally able to cross the BBB and this characteristic has inspired many research groups to try to develop efficient brain drug delivery systems. Immune cells are particularly sensitive towards inflammatory signals and would migrate towards injured tissue [35,36]. This includes inflammatory signals originating from the brain environment as well. Using a process called diapedesis, activated T-cells and macrophages interact with adhesion molecules on the surface of the BBB EC, thus allowing themselves to transmigrate in between microvascular EC junctions [37]. Mesenchymal stem cell (MSC) and neural stem cells (NSC) could also be used in cell-mediated delivery as they are able to home in on tumour locations [35]. Their tendency to migrate towards tissues in diseased states, or pathotropism, makes them a good candidate for a targeted therapeutic delivery vector into the brain. Their tumour tropism was found to be related to their abilities to detect and interact with extracellular matrix (ECM) that is produced by gliomas, various growth factors and cytokines, and the presence of chemokine receptors on the stem cells themselves [38].

In 2013, Batrakova et al. published a comprehensive review article on studies that investigated using cell mediated delivery into the brain [39]. In fact, the group themselves have reported promising preliminary results in their own study into using bone marrow derived macrophages to deliver catalase, an anti-inflammatory enzyme, into the brains of mice model for Parkinson’s disease [40]. Similarly, a 2010 study reported that macrophages that were engineered to deliver glial derived neurotrophic factor (GDNF) not only conferred neuronal protection, but also promoted axon regeneration in a mouse model for Parkinson’s disease [41].

However, the main downside of adopting this approach is the limitations with regards to the type of therapeutics that can be carried by these cells. Drugs that affect cellular biochemical pathways could lead to death of transporting cells before even reaching their intended diseased targets. Furthermore, stem cells and immunocytes could act on the drugs they carry, leading to premature offloading, or the drugs being metabolised thereby rendering them ineffective for treatment [24,39]. Moreover, macrophages of the M1 subtype in particular, are highly pro-inflammatory and as such could lead to neuronal damage when introduced into the brain environment [39].

### 2.4. Cell Penetrating Peptide

Therapeutics could also be conjugated to cell penetrating peptide (CPP), which is a special group of molecules made up of up to 30 amino acids that are intrinsically able to cross the cell membrane [42]. CPPs are typically proline- or arginine-rich moieties that when complexed with another moiety, confers amphipathicity, cationicity, or hydrophobicity to the assembly, thus enabling the complex to cross the cell membrane [42,43,44,45]. The mechanism by which CPPs use to enter cells is varied, depending on the peptides themselves. Pujals and Giralt have reported that proline-rich CPPs are internalised via the caveolae- and lipid-mediated endocytosis mechanism, while Futaki et al. discovered that arginine-rich CPPs relies on the micropinocytosis pathway [46,47].

A 2014 study by Srimanee et al. reported a novel CPP, called PepFect32, that was able to transport therapeutic plasmid DNA (pDNA) efficiently across in vitro BBB model [48]. Even more recently, Lakkadwala et al. improved liposomal cargo delivery into the brain by modifying the liposomes to express CPPs in the forms of either transactivated transcription (TAT) or the pentapeptide QLPVM, as well as the protein transferrin. Using this strategy, they were able to increase doxorubicin biodistribution in the brain by 10-fold compared to free drug administration [49]. The potential for CPPs to improve brain drug delivery is clearly promising, but selective delivery with this method is not guaranteed, especially if the CPP used is not a homing peptide and is able to interact non-specifically to cell membranes from any tissues [43]. In addition to this, introducing foreign protein into an unfamiliar tissue environment could set off inflammatory reactions, potentially leading to more complications.

### 2.5. Nanoparticle-Mediated Delivery

Studies have also been performed using nanoparticles (NPs) to assist in delivering therapeutics across the BBB. Some of the types of NPs used for this approach include metallic NPs, lipid NPs, polymeric NPs and targeted NPs [24,50,51]. Given that metallic NPs are too dense, drugs are not encapsulated in the particles and are instead conjugated on the surfaces of the NPs [24]. However, since they are a lot smaller than polymeric or lipid NPs, metallic NPs can travel across the BBB via CMT passive diffusion or trans-synaptic transport [24]. Polymeric NPs are typically large and can be used to encapsulate drugs, contrast agents as well as nucleic acids [50]. Studies have shown their ability to cross the brain EC via endocytosis, making them a valuable carrier for therapeutics that are otherwise too big to pass through the BBB [52]. Lipid NPs are made up of amphiphilic phospholipids similar to the ones that make up the cell membrane. Their compositional similarity to the cell membrane means that they are biocompatible and can be used to carry either hydrophobic or hydrophilic drugs [1]. Targeted NPs are modified such that they interact with a protein on the membrane surface to trigger uptake via RMT. For brain delivery purposes, various BBB-associated proteins including transferrin receptor (TfR) and insulin receptor (IR) have been studied to work with NPs to enable macromolecular drug trafficking [53].

Nevertheless, targeted therapy particularly involving metallic NPs have been shown to cause neurological toxicity leading to tissue damage and modifications as well as cognitive deficits [54]. Leite et al. has published a comprehensive review article on the adverse effects reported in over fifteen studies investigating complications that arose from introducing NPs into the brain environment [55]. Titanium oxide NP for instance was observed to affect mitochondrial function, triggering inflammation events in the brain, modifying synaptic plasticity and causing cognitive deficits [55,56,57]. Based on these reports, at this stage clinical safety is still a big concern in using NPs in brain delivery of therapeutics and should be addressed before treatments can be offered.

### 2.6. Monoclonal Antibodies as Molecular Chaperones

Monoclonal antibodies (mAb) are immunogenic proteins that can bind to specific epitopes of their targets and induce biochemical reactions. One strategy that has been developed is to use mAbs that target surface membrane proteins that allow the passage of large moieties through cerebrovascular EC and eventually into the brain tissue. mAbs that targets transmembrane proteins which are involved in RMT in the BBB, such as TfR and IR, have been widely studied to assist transcytosis of therapeutics into the brain [58].

Various groups in recent years have reported the validation of this strategy. Zhou and colleagues for instance has demonstrated that an anti-mouse transferrin antibody that is fused with the GDNF, cTfRmAb-GDNF, could be delivered to the brain in vivo [59]. Zhang and Pardridge similarly fused their anti-rat TfR Ab, OX26, to brain derived neurotrophic factor (BDNF) and have also demonstrated brain uptake in rats with middle cerebral artery occlusion, and simultaneously reduced stroke volumes in their test subjects [60]. In addition, the anti-mouse TfR antibody called 8D3 has also been studied for the trafficking of therapeutics in the form of polyethylene glycol (PEG) covered liposomes carrying the enzyme β-glucuronidase [61]. An anti-insulin receptor monoclonal antibody, 83-14, was also previously reported by Coloma and colleagues with results that suggest it can be used for assisting drugs to traverse the BBB [62].

To improve brain localisation of antibodies delivered via the bloodstream, several modification and functionalisation approaches have been developed. Adsorption-mediated transport (AMT) is one of the transport mechanisms that are present on the BBB that allows positively-charged molecules to interact with negatively-charged cell membrane and subsequently cross the BBB [63]. F(ab’)_2_ fragments of several antibodies have been previously shown to improve BBB transcytosis rates and brain accumulation [64,65,66]. However, there are a few disadvantages to this particular strategy. Since AMT is initiated by charge interactions between cell membrane and positively charged moieties, it is not a very specific delivery modality. Additionally, immunogenic peptides such as antibodies are still capable of triggering immune reactions in whatever environment they are introduced in [67].

Even though studies have shown somewhat promising results when using mAbs as molecular chaperones, giving access to foreign proteins, and especially a highly immunogenic group of molecules such as mAbs into the brain is a very risky proposition [68,69]. This has been reported extensively in clinical trials using mAbs to treat various brain diseases. For instance, bapineuzumab, an amyloid-β targeting mAb, has been reported to cause meningoencephalitis in 6% of patients in its phase II clinical trial, which led to the discontinuation of its phase III trials in 2012 [70]. Similarly, the phase II clinical trial of a peptide-based vaccine AN-1792 has also been terminated after reporting 15 out of 300 patients (5%) receiving the vaccine developed meningoencephalitis [71,72]. Furthermore, the presence of antibodies in the brain has also been linked to neurological disorders such as psychosis as reported by Pathmanandavel et al. in 2017 [73]. Another phase II trial involving the mAb cetuximab for glioblastoma treatment saw 5% of participants suffered from diminished consciousness within 24 h of administration [74]. Additionally, natalizumab was approved for use for the treatment of multiple sclerosis in 2006, but unfortunately it was later discovered that the mAb is linked to progressive multifocal leukoencephalopathy [75,76]. From these reports, it is clear that introduction of peptide-based molecules into the brain could lead to a host of adverse effects in patients and thus should be avoided.

Moreover, there are concerns about the reproducibility of antibody clinical trials, which put into question the potential of the antibodies tested as therapeutic agents [77,78]. In a 2015 article, Monya Baker suggested three factors that contributed to the problems of achieving consistent results in antibody trials; cross-reactivity, variability, and using the antibodies outside of manufacturers specifications [79]. Cross-reactivity is a situation in which the paratope, or antigen binding site of the antibody, can recognise similar antigen structures on different proteins. Antibodies capable of binding to multiple protein targets pose a serious risk of eliciting an immune response that could damage tissues and organs that are otherwise healthy. Since antibodies are raised in animals, the production of antibodies is subjected to batch-to-batch variability, which could potentially translate into inconsistent epitope recognition abilities, leading to varying experimental and trial results. The article also posited that the use of antibodies beyond manufacturers’ conditions could have also contributed to the repeatability crisis of antibodies. The proteins targeted by antibodies could have folded differently in various experimental and physiological conditions that could result in the obscuring of epitopes or changing of protein conformation that is crucial for antibody binding [79].

Following this, quality control of antibody production has to be more stringent and at the same time, thorough validation and characterisation studies must be completed before translational studies are performed with antibodies. That said, bearing in mind the requirement for animal hosts needed in the productions step coupled with market forces makes it a very expensive endeavour for manufacturers.

Despite all the BBB-crossing strategies listed above, issues of effectiveness, risks and costs involved with the available treatment methods are still concerns that need to be appraised. Invasive strategies of getting therapeutics into the brain introduce the risks of trauma, tissue injury and infection that could cause systemic complications. The non-invasive approaches to cross the BBB discussed here come with their own disadvantages as well. Therefore, there is a need to develop methods of delivering therapeutics efficiently while minimising risks of adverse effects.

## 3. Aptamer: The Antibody Alternative

In 1990, three groups in America separately worked on developing a new class of affinity ligands. Tuerk and Gold, Ellington and Szostak, and Robertson and Joyce, independently of each other published papers on an in vitro selection method of nucleic acid sequences that specifically bound to their respective targets [80,81,82]. Aptamers, as the sequences are called, are short single stranded nucleotide sequences that can bind to proteins or cells specifically and at high affinities [81]. They are also known as synthetic or chemical antibodies since aptamers are produced and developed without the need for a biological host [83]. The high specificity and binding affinity are the result of aptamers adopting unique secondary and tertiary structural conformations based on the nucleotide sequences themselves. Consequently, these conformations enable aptamers to interact with specific epitopes on the protein through ionic interactions, hydrogen bonding or van der Waals forces [84].

### 3.1. SELEX

**Figure 2:**
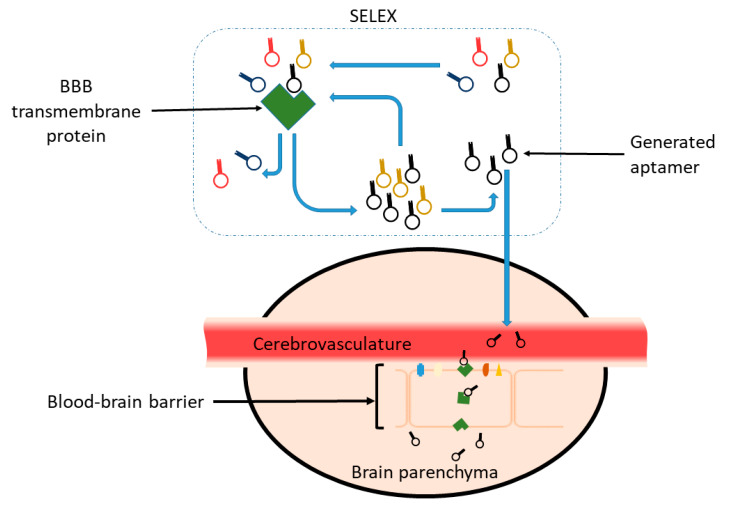
Aptamers generated against transmembrane proteins that are involved in transport mechanisms on the BBB could potentially overcome the restrictive nature of the barrier and make their way into the brain environment. Blue arrows show the chronological steps involved generating aptamers in protein SELEX. Figures adapted from references [2,86].

Aptamers are generated using an in vitro process called SELEX or Systematic Evolution of Ligands via Exponential Enrichment that was first developed by Tuerk and Gold in 1990 [80]. In SELEX (Figure 2), DNA or RNA libraries containing 10^13–15^ unique sequences are selected against a protein or cell of interest via an iterative in vitro process to obtain target-specific and strongly binding ligands [80,84,85,86,87]. DNA sequences are amplified by polymerase chain reaction (PCR) and later subjected to a binding reaction against the target [84,85]. Unbound nucleotide sequences are then removed from the total sequence population, and the bound sequences are amplified again by PCR to increase their copy number. Next, the newly generated library of nucleotide sequences is used in iterative SELEX cycles [86] and the cycle of binding reaction, separation and amplification is repeated as many times as required to promote the proliferation of aptamer species that have affinities to the target protein, while simultaneously weeding out low affinity sequences.

### 3.2. Aptamer Advantages

Aptamers have high thermostability and as such, following heat exposure, can revert to their original conformations and recover their specificity and affinity to their targets. This contrasts with antibodies which are made up of amino acids, and upon denaturation are predisposed to losing their original structure and hence their target binding abilities [88]. Aptamers are also immunologically inert which means that they are unlikely to instigate an immune reaction when introduced in a biological system [89]. This therefore minimises the risks of side effects from aptamers meant for therapeutic applications or other in vivo use.

Moreover, the small size of aptamers (~30 kDa for a 100 bp ssDNA) enables them to penetrate cells and tissues more efficiently [90]. With the increased reach, the target protein can be detected more comprehensively within the microenvironment. Being small molecules, aptamers are capable of moving rapidly through molecular and physical barriers where other affinity ligands such as antibodies could not [91]. As such, targeted delivery of therapeutics to a specific diseased site closed off by highly regulated structures can be completed effectively.

The generation and development of aptamers occur in vitro in a highly modifiable process. SELEX therefore obviates the need for biological organisms for aptamer maturation, unlike antibodies [92]. Consequently, in contrast to antibodies, aptamers do not suffer from batch-to-batch variation issues as they can be synthesised at high fidelity via oligonucleotide synthesis processes [93]. They are therefore much less technically demanding and can be generated easily at a much lower cost [87]. This would also mean that more rigorous tests to ensure aptamer specificity and performance could be executed at a lower cost. Additionally, since they are chemically synthesised, aptamer development methods are highly customisable to suit various assays and methods including surface plasmon resonance, capillary electrophoresis and nitrocellulose filter membrane, to name a few [94,95,96]. Hence, the aptamer generation process is an economical yet highly customisable method that can give us high yield of affinity ligands with excellent sequence fidelity.

Owing to their pH and thermostability, relatively simple biochemistry, and in vitro production methods, aptamers themselves are also highly modifiable. These modifications serve to improve either the binding affinity, stability or to functionalise them via conjugation to reporter molecules or therapeutic cargos [97,98,99]. This strategy allows for the development of targeted delivery of drugs as well as aptamer-based companion diagnostic assays.

### 3.3. Aptamer Limitations

Despite the advantageous features of aptamers, several notable limitations must be considered in order to develop aptamers for therapeutic applications. Since aptamers are made up of nucleotides, they are susceptible to digestion by nucleases especially in in vivo or in vitro tissue culture environments. Having said that, various strategies have been developed to improve aptamer nuclease resistance. For instance, the 2′OH of the sugar backbone of RNAs can be substituted with fluoro, amino or methoxy functional groups [97,100]. Aptamers can also be modified to incorporate locked nucleic acids (LNA), which is a special group of oligonucleotides with added methylene bridge between the 2′- and 4′- position of the ribose sugar [101]. Other modifications such as the addition of biotin or inverted thymidine at the 3′ end of aptamers would also increase aptamer resistance against 3′-exonucleases [102]. Moreover, a unique class of aptamers called spiegelmers, can be considered as well. Spiegelmers are not susceptible to ribonucleases as they are made out of enantiomers, or mirror images, of naturally occurring nucleotides, and are therefore not recognised by the enzymes as substrates [103].

Another approach to avoid nuclease degradation is by using aptamers that are made up of xenobiotic nucleic acid (XNA). In fully modified XNA aptamers, nonstandard nucleotides, such as α-l-threofuranosyl nucleic acid (TNA), 1,5-anhydrohexitol nucleic acid (HNA), or 2′-fluoro-arabinonucleic acid (FANA), are incorporated into sequences that make up the aptamer library in an in vitro selection process called X-SELEX [104,105,106,107]. A 2019 study by Eremeeva et al. reported that their rat vascular endothelial growth factor 164 (rVEGF164) XNA aptamers survived 72 h of incubation at 37 °C in 95% human serum, while their DNA aptamers counterparts were observed to be degraded after 24 h [105]. Similarly, a TNA aptamer that targets the human immunodeficiency virus reverse transcriptase (HIV-RT) developed by Dunn and colleagues was observed to be able to resist degradation after 8 h incubation at 37 °C in the presence of a potent 3′-exonuclease, snake venom phosphodiesterase (SVPE). This is in contrast to the HIV-RT DNA aptamer R1T, which degraded within minutes in the same assay [104].

Depending on their lengths, aptamers typically have a size of about 10–30 kDa which is up to 6 times smaller than a mAb [90,108]. Consequently, aptamers upon administration into the blood circulation have a higher renal clearance rate than antibodies. This characteristic, nevertheless, is not an inherently negative trait, as it depends on the use of the particular aptamer. For example, aptamers that target ubiquitous proteins for cytotoxic payload delivery might be better off being expelled from the circulatory system shortly after administration to minimise off-target side effects. Additionally, since aptamers are small, they exhibit better tissue penetration and could reach their target more effectively before being cleared by the kidney [90].

Nevertheless, if aptamer tissue penetration is lacking, conjugating the aptamer with large molecular weight compounds such as cholesterol will increase the size of the affinity ligand and prevents it from being filtered through the glomerulus thereby retaining it in the circulation for much longer [109,110].

Unlike antibodies, which are made up amino acids, aptamers are made up of four nucleotides (guanine, G, cytosine, C, adenosine, A, and tyrosine, T, or uracil, U). The relatively simple chemical structure of the nucleotides, which lack side chains and complex functional groups, means that aptamers possess limited structural diversity that could have otherwise enabled them to better interact with their targets [111]. In order to provide aptamers peptide-like features, Gold et al. have developed a strategy called slow off-rate modified aptamer or SOMAmer [112]. In this technique, the nucleotide deoxyuridine triphosphate is modified to include amide linkages at the 5′ position to simulate side chains typically found on proteins. The modifications have been shown to improve aptamer binding affinities and the amides help limit the rotation of the linkage bond and adds supplementary hydrogen bonding partners [97,98,111].

In SELEX, amplification of ssDNA sequences is a crucial step in order to increase the population of protein-binding sequences within the library. This is commonly performed using PCR, as it is the most practical and cost-efficient method to increase nucleotide sequences. However, this reaction also imposes a selection pressure on the DNA library, leading to PCR bias. The amplification efficiency of the sequences in the library depends on the sequences’ proclivity to form template-template hybrids, the correct annealing of primers and interference by PCR by-products [112]. Therefore, regardless of the binding affinity between a candidate aptamer and its target protein, its population frequency in the library may still be affected by its PCR efficiency. With that in mind, techniques have been developed to minimise PCR biases. Emulsion PCR involves adding mineral oil to the PCR mixture followed by vigorous vortexing to create microbubbles containing the PCR reaction which are separated from one another [113,114]. This approach helps by compartmentalising reactions such that a single or limited number of template DNA is enclosed in each reaction bubble, thereby preventing by-products, primer-dimers or product-product hybrids from interfering the amplification reactions of other templates [114].

Table 1 highlights the benefits and disadvantages of using aptamers as a molecular tool. Aptamers are a very exciting class of affinity ligands due to their robustness, malleability, and lack of immunogenicity. Studies exploring their practicality in drug delivery strategies should be promoted to provide us with alternative cutting-edge approaches in medical treatments and diagnostics.

### 3.4. Reported Aptamers That Could Potentially Cross the BBB

Aptamers, owing to the characteristics they possess, could therefore be very useful for transporting therapeutics into the brain. Being small, modifiable, thermostable, and non-immunogenic molecules allows for the development of more efficient and drug transport strategies that can overcome the BBB. Considering this, several groups have developed aptamers that target the BBB EC or proteins on its membrane to negotiate a way across the BBB. In this section, these reported aptamers will be briefly discussed to illustrate the potential of aptamers.

#### 3.4.1. GS24, DW4, TfRA4 & TEPP Aptamer

In 2008, Chen generated a DNA aptamer against the extracellular domain of mouse TfR called GS24 and highlighted its ability to internalise into mouse fibroblast cells [115]. The work on GS24 aptamer was continued further by Porciani et al., which performed modifications on the original aptamer to produce new aptamer versions with higher binding affinities called GS24min and DW4. The authors discovered that the truncated GS24 aptamer, GS24min was not able to bind to human TfR, but DW4 has an affinity towards both mouse and human TfR expressed on mouse fibroblast cell line NIH3T3 and human pancreatic cell cancer line MIA PaCA-2, respectively [116]. Macdonald and colleagues performed their own aptamer modification experiments on the GS24 aptamer and generated a novel TfR aptamer called TfRA4. The TfRA4 aptamer has a much improved binding affinity and for the first time was observed to be internalised by the mouse brain endothelial cell line, bEnd.3 [117]. Building up from this finding, the group then developed a bifunctional aptamer construct in which the TfRA4 aptamer is conjugated with another aptamer called EpA, which specifically targets epithelial cell adhesion molecule (EpCAM) [91,118]. For this study, EpCAM was chosen as the other transmembrane protein target due to its overexpression in solid tumour cells [119,120]. The resulting aptamer, called TEPP, has been shown to be able to not only internalise, but to also traverse across in vitro BBB model [118,121]. Additionally, when introduced into a mouse model of triple-negative breast cancer brain metastases, accumulation was observed in the tumour cells [118].

#### 3.4.2. R11-3 & R39 Aptamer

A Korean group has published a paper on developing a BBB-crossing aptamer with human and mouse cross-reactivity using cell SELEX. Pooja et al. reported that the R11-3 aptamer could be internalised by both immortalised and primary EC lines from either mouse or human. The authors then truncated the R11-3 aptamer down to 39 nucleotide long, dubbed it R39, and conjugated it to short and long interference RNA that targets the vascular endothelial growth factor receptor 2 (VEGFR2) mRNA. In vitro administration of the R39-liRNA (long interfering RNA) complex to the human cardiac microvascular endothelial cell, hCMEC/D3, showed 65% knockdown of the VEGFR2 mRNA, even without using a lipid-based transfection reagent to assist with payload delivery [122]. However, it is unknown which particular protein on the microvascular EC that is being targeted by the R39 aptamer. Further studies are needed to identify the target, and to determine if the aptamer is suitable for use in brain pathologies.

#### 3.4.3. A15 Aptamer

In 2013, Cheng et al. attempted to isolate and develop RNA aptamers that are able to cross the BBB without specifically nominating their target proteins [123]. This was performed using a type of selection process called in vivo SELEX. In this study, the group administered an injection to the mouse tail containing single-stranded RNA (ssRNA) sequences. To assist with in vivo survivability, the ssRNA sequences were modified to incorporate 2-fluoronuclear triphosphates. After one- and three-hours post injection, the brain was harvested and RNAs were recovered before amplifying them via real time reverse transcription-PCR (qRT-PCR). The SELEX cycle was repeated for up to 22 rounds. Using this approach, they managed to identify an RNA aptamer sequence called A15. Although the exact target of the aptamer was not identified, the authors’ in situ hybridisation results further confirmed its ability to penetrate the BBB [123]. That said, since the aptamer was developed in a mouse model, it remains to be seen if the aptamer is capable of crossing the BBB made up of human EC.

#### 3.4.4. C2.min and Waz Aptamers

The human TfR-binding RNA aptamer C2.min was generated in 2012 by Wilner and colleagues using a combination of protein and cell SELEX methods. It should be noted that while the aptamer was specifically developed against human TfR and cell internalisation was observed, the authors used the HeLa cervical tumour cell line, as opposed to BBB EC cell lines in their study [124]. Waz, the other human TfR RNA aptamer developed by the same group, had a better dissociation constant than C2.min and cell internalisation was observed in their binding assay study. However, the group used a T lymphocyte Jurkat cell line, and not BBB EC, to demonstrate aptamer uptake [125]. Investigations into their internalisation and transcytosis capabilities should be investigated further against cerebrovascular EC to confirm their potential in crossing the BBB.

#### 3.4.5. GL21.T Aptamer

In 2012, Cerchia and colleagues developed an aptamer called GL21.T that could bind to Axl, which is a tyrosine receptor kinase with established roles in tumorigenesis and overexpression in tumour cells [126]. The Italian group originally set out to use the GL21.T aptamer to disrupt Axl activity and therefore halt cancer progression. Follow up work by the same group in 2016 revealed that the GL21.T could move across a tri-culture in vitro BBB model in either its original aptamer form or when it is complexed with therapeutic miRNAs [127]. The authors posited that GL21.T was able to move across the in vitro model by interacting with Axl expressed on EC and pericytes, to then migrate towards the basolateral compartment of the assay. A point of concern, however, is the fact that upon introduction of the aptamer, barrier permeability increased, and it took 6 h for the in vitro model to recover its permeability profile. As mentioned in Section 2.1 on one of the limitations of FUS, BBB leakiness could allow non-specific entry of foreign biologicals or pathogens into the brain causing undesired side effects. It remains to be seen if this observation holds true for in vivo models as well, since there has not been a published follow up study on this topic to date.

#### 3.4.6. Gint4.T Aptamer

The Gint4.T aptamer was originally developed by Camorani et al. in 2014 as a therapeutic modality against glioblastoma (GBM). The 33 nt RNA aptamer has an affinity towards the ectodomain of the human platelet-derived growth factor receptor β (PDGFRβ), which is overexpressed in glioma stem cells and has been previously linked to tumour growth [128,129]. The group reported that in their in vivo study, Gint4.t was able to hinder GBM proliferation and migration. They suggested that the aptamer works by inhibiting PDGFRβ activity, which has been documented to be crucial for tumorigenesis. The authors noted that by itself, the aptamer could not cross the BBB [130]. However, in a follow up article published by Monaco and colleagues, conjugation of the Gint4.T aptamer with polymeric nanoparticle (PNP) allowed the complex to penetrate the BBB in vivo [50]. Interestingly, while the aptamer-PNP exhibited transcytosis properties, a scrambled sequence-PNP complex could not cross the BBB suggesting that both the Gint4.T aptamer and the PNP are needed to traffic themselves across the BBB. Development of this aptamer was carried forward by another group from China in 2019. Shi et al. took the modifications further of the Gint4.T aptamer by conjugating it to another aptamer called GMT8, which targets the U87 glioma cell line, and another novel nanocarrier called tetrahedral framework nucleic acid (tFNA) [131]. In their experiment, they have shown that the construct used was able to cross an in vitro BBB model and target in the U87 cell line, highlighting its potential for targeted drug delivery into glioblastoma [131].

#### 3.4.7. IR-A48 and GL56 Aptamers

Insulin receptor (IR) is one of the transmembrane proteins involved in RMT that can be found on the BBB [3]. To date, two IR-targeting aptamers, IR-A48 and GL56, has been reported by two research groups from South Korea and Italy, respectively [132,133]. The IR-A48 aptamer was found to bind to IR at a very high affinity and interestingly was able to internalise into 3T3-L1 adipocyte cell line [132]. Similarly, the GL56 aptamer can also be internalised upon incubation with the U87MG human glioblastoma cell line and demonstrated inhibitory properties against the IR signalling pathway which led to the reduction of cell viability [133]. Although in their study Iaboni and colleagues used the RNA aptamer against a glioblastoma cell line to examine its internalisation capability, IR is also highly expressed on the BBB and has been shown to undergo endogenous transcytosis [89,134,135]. Moreover, Coloma et al. has reported that IR can be used as a target for monoclonal antibodies to help therapeutics traverse the BBB which suggests that a similar approach using aptamers could work as well [62]. That said, validation studies of that are still required since neither IR-A48 nor GL56 has been investigated in in vitro or in vivo BBB transcytosis experimental setups.

#### 3.4.8. RNV-L7 Aptamer

Low-density lipoprotein receptor (LDL-R) is a cell surface protein that moderates the uptake of LDLs into cells and has been shown to be expressed on luminal and abluminal surfaces of BBB EC [136]. Recently, Wang et al. reported on the generation of RNV-L7 aptamer that specifically targets LDL-R. In their study, the group conjugated the aptamer with DNAzyme to inhibit miR-21 expression in Huh-7 liver cancer cells using their aptamer-DNAzyme construct [137]. The authors reported have reduction of miR-21 expression using the complex, which suggests that it was able to take advantage of the LDL-R pathway to gain entry into the liver cancer cells [137]. It will be interesting to see if the same aptamer could internalise and transcytose across BBB and assist with therapeutic delivery as well.

Even though a handful of aptamers that can cross the BBB have been studied by various groups (Table 2), they are still in their early development stages. The potential for aptamers as an efficient and targeted BBB crossing agent has to be explored further. As discussed in Section 2.2, current therapeutic delivery strategies, while promising in certain aspects, come with their own disadvantages. Aptamers are good candidates for shuttling therapeutics from the circulatory system and into the brain tissue especially due to its specificity, flexibility, and low immunogenicity.

### 3.5. Alternative Aptamer Targets

In addition to continuing research with the aptamers that have been mentioned in Section 3.4, other protein targets could be investigated as the key to BBB therapeutics delivery via endogenous transport mechanisms. To date, majority of studies negotiating a way of crossing the BBB via transmembrane proteins have focused on the TfR protein, IR or using either human or mouse microvascular endothelial cell lines to select for either aptamers or antibodies [24,122,123,138]. However, there are other transmembrane proteins that have not been given much attention. These proteins could potentially lead to the development of novel aptamers capable of crossing the BBB efficiently and assist in shuttling therapeutic agents into the brain.

In a recent paper by Zuchero et al., it was reported that several novel transmembrane proteins are also selectively expressed on the BBB EC. Among these are the glucose transporter (Glut1), cluster of differentiation 98 (CD98) and basigin [139]. Glut1 has the crucial role of trafficking glucose as an energy source for brain tissue, which explains their abundance on the BBB EC [139]. The CD98 protein forms a heterodimer complex with another transmembrane protein called L-type amino acid transporter 1 (LAT1) that allows the transport of large neutral amino acids between the brain and blood environments [140]. Meanwhile, basigin is a member of the immunoglobulin superfamily and is able to interact with different molecules, depending on where it is expressed, leading to various downstream pathways [141]. In addition to those proteins, leptin hormone receptor (LepR), and specifically leptin receptor subtype a and c (LepRa and LepRc) have also been reported to be highly expressed in the BBB [142,143]. These BBB surface protein candidates should be considered as targets for the generation of novel aptamers that could potentially traffic therapeutics across the BBB.

## 4. Conclusions

Delivering drugs into the brain environment is a challenge due to the nature of the BBB anatomy, and as a result, complicates the treatments of diseases of the brain such as primary and secondary brain tumours, Alzheimer’s disease, and multiple sclerosis. Different strategies of trying to cross the BBB and deliver therapeutics to the brain parenchyma come with their own benefits and disadvantages. In light of that, there is a need to develop modalities that allows for efficient and tissue-specific delivery of therapeutics into the brain. This review article posits that aptamers should be explored further in terms of their potential to negotiate access across the BBB and assist in tissue-specific therapeutics delivery. The lack of immunogenicity in particular is one of aptamers’ most noteworthy features, especially since immunogenic reactions in the brain environment could negatively affect healthy tissues in the CNS, which could lead to serious complications.

A number of published aptamers should be investigated further on their BBB-traversing abilities and follow-up studies investigating the functionalisation of them should be performed. This article has also discussed the transmembrane proteins present on the cerebrovascular EC, which could be key candidate targets for future aptamer generation and development studies. Considering how various brain pathologies can have different clinical manifestations, having many options for therapeutic modalities could allow for development of more effective treatments.

## Figures and Tables

**Table 1:** Summary of aptamer advantages and limitations.

Advantages	Limitations
Small size	High clearance rate
Cheap	Susceptible to nuclease degradation
Highly modifiable	Limited building block diversity
Thermostable	PCR bias in SELEX method
Immunologically inert Easily manufactured with high reproducibility	

**Table 2:** Studies that have been completed on aptamers with the potential to assist in BBB transcytosis of therapeutics.

Aptamer Name	Target	Internalisation/Transcytosis/Brain Localisation	References
A15	Mouse BBB EC	Localisation observed in vivo	[123]
C2.min & Waz	Human TfR	Internalisation observed for cervical tumour and T lymphocyte cell lines, no data for BBB EC	[124,125]
Gint4.T	PDGFRB	Transcytosis observed in vitro	[50,130,131]
GL21.T	Axl	Transcytosis observed in vitro	[126,127]
GL56	IR	No experimental confirmation	[133]
GS24, GS24min & DW4	Mouse TfR (GS24 & GS24min), human and mouse TfR (DW4)	Internalisation observed in mouse fibroblast cell line NIH3T3 and pancreatic carcinoma cell line MIA PaCa-2, no data for BBB EC	[115,116]
IR-A48	IR	No experimental confirmation	[132,133]
R11-3 & R39	Human and mouse BBB EC	Internalisation observed in vitro, no confirmation for transcytosis	[122]
RNV-L7	LDL-R	No experimental confirmation	[137]
TfRA4 & TEPP	TfR (TfRA4), TfR & EpCAM (TEPP)	Transcytosis and brain localization observed in vitro and in vivo	[117,118,121]

Axl = tyrosine kinase receptor, BBB EC = blood-brain barrier endothelial cells, EpCAM = epithelial cell adhesion molecule, IR = insulin receptor, LDL-R = low-density lipoprotein receptor, PDGFRB = platelet-derived growth factor receptor beta, TfR = transferrin receptor
